# Breast Cancer Mortality vs. Exercise and Breast Size in Runners and Walkers

**DOI:** 10.1371/journal.pone.0080616

**Published:** 2013-12-09

**Authors:** Paul T. Williams

**Affiliations:** Life Sciences Division, Lawrence Berkeley National Laboratory, Berkeley, California, United States of America; University of Nebraska Medical Center, United States of America

## Abstract

**Purpose:**

Identify predictors of breast cancer mortality in women who exercised below (<7.5 metabolic equivalent hours/week, MET-hours/wk), at (7.5 to 12.5 MET-hours/wk), or above (≥12.5 MET-hours/wk) recommended levels.

**Methods:**

Cox proportional hazard analyses of baseline pre-diagnosis MET-hours/wk vs. breast cancer mortality adjusted for follow-up age, race, baseline menopause, and estrogen and oral contraceptive use in 79,124 women (32,872 walkers, 46,252 runners) from the National Walkers' and Runners' Health Studies.

**Results:**

One-hundred eleven women (57 walkers, 54 runners) died from breast cancer during the 11-year follow-up. The decline in mortality in women who exercised ≥7.5 MET-hours/wk was not different for walking and running (P = 0.34), so running and walking energy expenditures were combined. The risk for breast cancer mortality was 41.5% lower for ≥7.5 vs. <7.5 MET-hours/wk (HR: 0.585, 95%CI: 0.382 to 0.924, P = 0.02), which persisted when adjusted for BMI (HR: 0.584, 95%CI: 0.368 to 0.956, P = 0.03). Other than age and menopause, baseline bra cup size was the strongest predictor of breast cancer mortality, i.e., 57.9% risk increase per cup size when adjusted for MET-hours/wk and the other covariates (HR: 1.579, 95%CI: 1.268 to 1.966, P<0.0001), and 70.4% greater when further adjusted for BMI (HR: 1.704, 95%CI: 1.344 to 2.156, P = 10^−5^). Breast cancer mortality was 4.0-fold greater (HR: 3.980, 95%CI: 1.894 to 9.412, P = 0.0001) for C-cup, and 4.7-fold greater (HR: 4.668, 95%CI: 1.963 to 11.980, P = 0.0004) for ≥D-cup vs. A-cup when adjusted for BMI and other covariates. Adjustment for cup size and BMI did not eliminate the association between breast cancer mortality and ≥7.5 MET-hour/wk walked or run (HR: 0.615, 95%CI: 0.389 to 1.004, P = 0.05).

**Conclusion:**

Breast cancer mortality decreased in association with both meeting the exercise recommendations and smaller breast volume.

## Introduction

Prior studies suggest that physical activity reduces breast cancer risk by about 25 percent [1]. This estimate is based primarily on studies that measure cumulative energy expended from all physical activities regardless of intensity [1], [2]. Physical activities that expend 3- to 6-fold the energy expended by sitting at rest (3 to 6 metabolic equivalents or METs, 1 MET = 3.5 ml O_2_•kg^−1^•min^−1^) such as walking are classified as moderate intensity, those that expend ≥6 METs such as running are classified as vigorous, and <3 METs as light [2], [3]. Few studies have compared the effects of moderate and vigorous exercise in general, or specific exercises in particular, on breast cancer risk [2].

It is unclear whether breast cancer risk factors apply to all women equally. In young, lean women, larger breast size has occasionally been associated with greater breast cancer risk [4], [5]. Running and walking promote weight loss and attenuates middle-age weight gain [6], [7], and as a group, women runners in particular are leaner than sedentary women [8]. Whether the leanness of women runners and walkers maintains the importance of their breast size as a risk factor later in life remains to be determined. It is also possible that leanness was indicative of being physically active in previous studies showing a breast cancer-breast volume association [4], [5].

This paper examines the dose-response relationships of breast cancer mortality to baseline pre-diagnosis exercise dose and bra cup size in a large cohort of women runners and walkers. There are several potential advantages to the focusing specifically on running and walking rather than all types of exercise. Running and walking occur in discrete bouts, and particularly for running, must be done regularity to be sustained. In addition, running and walking exercise energy expenditures can be calculated from weekly distance run or walked, which appears to be a better metric for studying dose-response than the traditional time-based calculations used by other studies [9]–[11]. In particular, we have shown that the associations of body weight, diabetes, hypertension, and high cholesterol with distance-based estimates of energy expenditure were approximately two-fold larger than those observed with time-based estimates [9]–[11]. This is important because non-differential errors in recall of physical activity are likely to bias results toward the null hypothesis in most existing studies [12], and measurement error associated with other physical activity questionnaires may substantially underestimate the impact of physical activity on cancer risk [13]. Women who run or walk for exercise tend to be leaner than the general population, which may affect whether breast size is a risk factor for breast cancer mortality.

## Materials and Methods

The National Walkers' and Runners' Health Studies have been described previously [6]–[11], [14]–[16]. Walkers were recruited between 1999 and 2001, while runners were recruited in two waves, between 1991 and 1993 (phase I) and between 1998 and 2001 (phase II). The runners and walkers were recruited using the same questionnaire (modified slightly for the different activities), using the same sampling domain (subscription lists to running and walking publications, running and walking events), and using the same survey staff. Questionnaires were distributed by mail or in event packets and were obtained only at baseline. Distance run was obtained from the question “Average miles run per week for ” and then listed the current and preceding five years with spaces for the responses (the most recent distance was used for analyses). Pace was determined by the question “During your usual run, how many minutes does it take for you to run one mile?” Walking distance and pace were ascertained using the same questions for walking instead of running. Walking energy expenditure (MET-hours/wk) was computed by converting the reported usual weekly distance into duration (i.e., distance/mph) and then calculating the product of the average hours walked per week and the MET value corresponding to their reported pace [9]. Running MET values were calculated as 1.02 MET•hours per km [10]. Previously, we have reported strong correlations between repeated questionnaires for self-reported running distance (r = 0.89) [14]. The study protocol was approved by the University of California Berkeley committee for the protection of human subjects, and all subjects provided a signed statement of informed consent. The data are available pending human use approval.

Height and weight were determined by asking the participant, “What is your current height (in inches, without shoes)?” and, “What is your current weight (pre-pregnancy weight if pregnant)?” Body mass index (BMI) was calculated as weight in kilograms divided by the square of height in meters. Self-reported waist, hip, and chest circumferences were elicited by the question, “Please provide, to the best of your ability, your body circumference in inches: waist___, hip___, and chest___,” without further instruction. Elsewhere, we have reported the strong correlations between self-reported and clinically measured heights (r = 0.96) and weights (r = 0.96) [14]. Self-reported waist, hip and chest circumferences were somewhat less precise, as indicated by their correlations with reported circumferences on a second questionnaire (r = 0.84, r = 0.79, r = 0.93, respectively) and with their clinical measurements (r = 0.68, r = 0.63, r = 0.77, respectively) [14]. Self-reported bra cup sizes were coded on a 5-point scale: 1 (A cup), 2 (B cup), 3 (C cup), 4 (D cup), and 5 (E cup or larger). Split cup sizes were coded as an intermediate value (e.g., BC = 2.5) when analyzed as a continuous scale, and assigned to the lower cup size when analyzed by categories.

Intakes of meat, fish and fruit were based on the questions “During an average week, how many servings of beef, lamb, or pork do you eat”, and “…pieces of fruit do you eat”. Alcohol intake was estimated from the corresponding questions for 4-oz. (112 ml) glasses of wine, 12-oz. (336 ml) bottles of beer, and mixed drinks and liqueurs. Alcohol was computed as 10.8 g per 4-oz glass of wine, 13.2 g per 12 oz. bottle of beer and 15.1 g per mixed drink. Correlations between these responses and values obtained from 4-day diet records in 110 men were r = 0.46 and r = 0.38 for consumptions of meat and fruit, respectively. Family history of breast cancer was based on whether the respondent had a mother or sister who had breast cancer before age 55 (phase I), and from listing of cancers by site in all first-degree relatives (phase II). For consistency, the phase I definition of family history was used for all participants.

Mortality surveillance was completed through December 31, 2008 using the National Death Index [17]. Cox proportional hazard analyses (JMP version 5.1, SAS Institute, Cary SC) were used to test whether breast cancer deaths (International Classification of Disease version 9 codes 174–175 and version 10 code C50) were significantly related to MET-hours/wk run or walked and other risk factors when adjusted for follow-up age (age and age^2^ at death or end of follow-up), race, menopause, and oral contraceptive and estrogen use. The covariates were selected for their significant relationship with breast cancer mortality. Results are presented as hazard ratios (HR) and their percent risk reduction {calculated as 100*(HR-1)} for categories of running energy expenditure relative to falling short (<7.5 MET-hours/week), achieving (7.5 to 12.5 MET-hours/week), or exceeding the minimum exercise energy expenditure recommended for health (>12.5 MET-hours/week) [3]. All analyses exclude women who reported a previous breast cancer diagnosis on their baseline questionnaire or who survived less than one year from their baseline survey.

## Results

One-hundred eleven (57 walkers, 27 runners from the first recruitment phase, and 27 runners from the second recruitment) of the 79,124 runners and walkers died from breast cancer as the underlying cause of death during (mean±SD) 11.0±2.11 years of mortality surveillance. Tables 1 and 2 present the sample characteristics by baseline running energy expenditure (MET-hours/wk) and bra cup size.

**Table 1:** Sample characteristics by MET-hours/week/d run.

	MET-hours/week run or walked†			
	<7.5	7.5 to 12.5	12.5 to 25.0	≥25.0
Sample size	12,641	9,137	25,352	31,994
Runners (%)	27.91	28.23	52.30	84.03
Follow-up age*	56.92±14.27	56.66±13.50	54.53±12.37	50.89±11.09
Follow-up (years)	9.55±1.99	9.69±1.95	10.41±2.43	11.41±2.86
Smokers (%)	6.47	4.51	3.62	2.36
Exercise (MET-hour/wk)	3.52±2.34	10.10±1.57	19.35±3.92	45.74±17.50
Education (years)	15.15±2.99	15.40±2.53	15.60±2.56	15.83±2.44
Fruit (pieces/day)	1.33±1.06	1.49±1.09	1.53±1.32	1.62±1.15
Meat (servings/day)	0.40±0.37	0.36±0.35	0.32±0.32	0.24±0.46
Alcohol (g/d)	4.99±9.98	5.86±10.27	6.64±10.31	6.95±10.81
BMI (kg/m^2^)	26.62±6.47	25.00±5.00	23.42±4.03	21.60±2.90
Body circumferences				
Chest (cm)	95.20±10.08	93.10±8.28	90.92±6.93	88.31±5.48
Waist (cm)	80.81±14.00	77.39±11.53	73.68±9.50	69.41±7.24*
Hips (cm)	101.51±13.50	98.74±10.95	95.66±9.00	91.30±7.38
Bra cup size ‡	2.65±0.96	2.51±0.93	2.32±0.91	2.02±0.85
A-cup (column percent)	11.32	13.54	18.91	28.99
B-cup (column percent)	34.29	38.60	41.44	45.68
C-cup (column percent)	32.96	31.26	28.35	19.70
≥D-cup (column percent)	21.43	16.60	11.30	5.63
Age menarche (year)	12.62±1.84	12.70±1.77	12.79±1.66	12.96±1.64
Menopausal (%)	45.59	42.55	33.41	22.09
Nulliparous (%)	32.18	31.86	36.83	47.80
Age first pregnancy	24.17±5.17	24.51±5.04	24.90±4.98	25.30±5.03
Breast fed (months)	6.29±12.88	6.72±13.00	6.86±13.41	6.15±12.79
Family history	10.28	9.77	8.96	7.97
Oral contraceptives (% at baseline)	12.39	13.54	16.32	19.91
Estrogen (%)	13.26	13.08	10.81	6.61
Estrogen/progesterone (%)	10.82	11.92	10.68	7.25

* Calculated as the age at death or December 31, 2008. †All variables show an association with MET-hours/week at P≤0.05. ‡ coded A = 1, B = 2, C = 3, D = 4, ≥E = 5.

**Table 2:** Baseline sample characteristics by breast volume.

	Self-reported bra cup size†			
	A	B	C	D
Sample size	14,818	29,072	17,975	7,739
Runner (%)	79.46	65.43	47.89	28.88
Follow-up Age*	51.29±10.73	52.88±12.50	54.66±12.92	56.73±13.03
Follow-up (years)	11.36±2.90	10.79±2.66	10.20±2.34	9.75±2.00
Exercise (MET-hours/wk)	34.04±22.58	28.29±20.16	22.22±17.34	17.29±14.90
Education (years)	16.02±2.34	15.71±2.65	15.43±2.61	15.14±2.67
Smokers (%)	2.42	3.33	4.04	5.31
Fruit (pieces/day)	1.59±1.13	1.55±1.33	1.49±1.08	1.48±1.10
Meat (servings/day)	0.26±0.30	0.29±0.49	0.33±0.34	0.38±0.36
Alcohol (g/d)	6.55±9.97	6.62±10.44	6.50±10.82	5.56±10.70
BMI (kg/m^2^)	20.85±2.25	22.47±3.44	24.62±4.67	27.81±6.41
Body circumferences				
Chest (cm)	86.60±4.70	89.39±5.64	93.17±7.20	98.82±10.58
Waist (cm)	68.32±6.09	71.72±8.56	76.30±11.09	82.84±14.33
Hips (cm)	90.62±6.75	93.74±8.49	97.72±10.49	103.50±13.62
Age menarche (year)	12.97±1.58	12.89±1.70	12.72±1.72	12.48±1.75
Menopausal (%)	20.71	28.46	37.08	46.07
Nulliparous (%)	45.55	43.17	36.03	29.81
Age first pregnancy	26.08±4.82	24.99±5.04	24.36±5.02	23.79±5.07
Breast fed (months)	7.69±14.39	6.12±12.64	6.49±13.04	6.74±13.41
Family History (%)	8.63	8.25	9.43	9.47
Oral contraceptives (%)	18.77	18.81	16.47	11.31
Estrogen (%)	5.58	8.86	12.24	15.28
Estrogen/ progesterone (%)	7.40	9.40	10.80	11.92

* Calculated as the age at death or December 31, 2008. †All variables show an association with reported bra cup size at P≤0.05.

### Running and walking

When adjusted for follow-up age, race, menopause, oral contraceptive, and estrogen and estrogen/progesterone medication use, the decline in breast cancer mortality in women who met or exceeded the current physical activity recommendations was not significantly different for walking and running (P = 0.34). In addition, relative to <7.5 MET-hours/wk at baseline, the reduction in risk was similar for 7.5 to 12.5 MET-hour/wk and >12.5 MET-hour/wk for running and walking combined (Table 3). Thus, the risk for breast cancer mortality was 41.5% lower for ≥7.5 vs. <7.5 MET-hours/wk (HR: 0.585; 95%CI: 0.385 to 0.924, P = 0.02), which was unchanged when adjusted for baseline BMI (HR: 0.584, 95%CI: 0.368 to 0.956, P = 0.03). The inverse relationship between breast cancer mortality and MET-hours/d run or walked persisted when further adjusted for years of education, baseline smoking, intakes of meat, fruit, and alcohol, waist circumference, hip circumference, chest circumference, age of menarche, nulliperousness, age of first pregnancy, number of live births, months of breast feeding, or family history of breast cancer (all P≤0.05, results not displayed).

**Table 3:** Survival analyses for breast cancer mortality by reported exercise level.

MET-hours/wk run or walked	Cases/sample	Person-years mortality surveillance	Hazard ratio (95% Confidence interval)*	
			No adjustment for BMI	Adjusted for BMI
<7.5	28/12,641	118,764	1.0	1.0
				
7.5–12.5	10/9,137	87,657	0.498	0.471
			(0.230, 0.993)	(0.208, 0.973)
			P = 0.05	P = 0.04
				
>12.5	73/57,346	625,665	0.598	0.609
			(0.385, 0.953)	(0.378, 1.012)
			P = 0.03	P = 0.06

* Adjusted for follow-up age (age, age^2^), race, menopause, and oral contraceptive and estrogen or estrogen/progesterone use. Additional adjustment for BMI as indicated.

### Breast volume

Baseline breast volume, as indicated by bra cup size, was inversely related to MET-hour/wk run or walked (Table 2). Other than age and menopause, baseline bra cup size was the strongest predictor of breast cancer mortality, i.e., 57.9% greater risk (HR: 1.579, 95%CI: 1.268 to 1.966, P = 0.0001) per increment in cup size when adjusted for MET-hours/wk and the other covariates, and 70.4% greater risk (HR: 1.704, 95%CI: 1.344 to 2.156, P = 10^−5^) when also adjusted for BMI. Table 4 shows that not only did adjustment for BMI not explain the association, BMI adjustment actually increased the hazard ratios. In addition, the concordance between breast cancer mortality and bra cup size persisted when further adjusted for years of education, baseline smoking, intakes of meat, fruit, and alcohol, waist circumference, hip circumference, chest circumference, age of menarche, nulliperousness, age of first pregnancy, number of live births, months of breast feeding, or family history of breast cancer (all P≤0.0001, results not displayed). Reported bra cup size at age 18 was also significantly related to breast cancer mortality (HR: 1.363 per cup size, 95%CI: 1.050 to 1.751, P = 0.02) but not when adjusted for baseline cup size (P = 0.53), whereas baseline cup size remained predictive of breast cancer mortality when adjusted for cup size at 18 (HR: 1.521 per increment in cup size, 95%CI: 1.183 to 1.952, P = 0.001). Moreover, MET-hours/wk run or walked, follow-up age, baseline nulliparousness, baseline menopause status, and type of exercise (runners vs. walkers) did not significantly affect the bra cup-breast cancer relationship (P = 0.12, P = 0.26, P = 0.91, P = 0.86, and P = 0.42, respectively, for their interactions). Adjustment for bra cup size and BMI in addition to the other standard covariates did not eliminate the association between lower breast cancer mortality and achieving or exceeding the exercise recommendations (HR: 0.615, 95%CI: 0.389 to 1.004, P = 0.05).

**Table 4:** Survival analyses for breast cancer mortality by breast volume (bra cup size).

Bra cup size	Cases/sample	Person-years mortality surveillance	Hazard ratio (95% Confidence interval)*	
			No adjustment for BMI	Adjusted for BMI
A cup	8/14,818	167,565	1.0	1.0
				
B-cup	30/29,072	311,629	1.848	1.890
			(0.885, 4.342)	(0.900, 4.458)
			P = 0.11	P = 0.10
				
C-cup	37/17,975	181,752	3.516	3.980
			(1.694, 8.246)	(1.894, 9.412)
			P = 0.0004	P = 0.0001
				
≥D-cup	19/7,739	74,697	3.929	4.668
			(1.727, 9.771)	(1.963, 11.980)
			P = 0.0009	P = 0.0004

* Adjusted for follow-up age (age, age^2^), MET-hours/wk run or walked, race, menopause, and oral contraceptive and estrogen or estrogen/progesterone use. Additional adjustment for BMI as indicated.

## Discussion

Public health guidelines recommend that adults “should do at least 150 minutes (2 hours and 30 minutes) a week of moderate-intensity, or 75 minutes (1 hour and 15 minutes) a week of vigorous-intensity aerobic physical activity, or an equivalent combination of moderate- and vigorous-intensity aerobic activity” [2]. Our data suggest the risk for breast cancer mortality is reduced substantially by meeting the recommendations. The 41.5% reduction for breast cancer mortality for ≥7.5 MET-hours/wk could not be explained by other purported breast cancer risk factors, including BMI and regional adiposity. The reduction in mortality was not exclusive to either premenopausal or postmenopausal disease. The substantial risk reduction we observed for breast cancer mortality may also reflect, in part, the superiority of calculating MET-hours/wk from distance run than from time spent exercising [9]–[11]. The 30% reduction in breast cancer mortality that has occurred between 1991 and 2009 in the United States [18] suggests the opportunity for additional reductions in mortality through increased exercise participation.

The reduction in breast cancer mortality with running and walking we observed could be due to reduced risk for incident breast cancer, improved survival in women diagnosed with breast cancer, or both. Our data revealed no significant difference in the reduction in breast cancer mortality between running and walking. However, the power to detect small differences in risk was probably limited. Although the protective effects of physical activity on breast cancer risk are reasonably well-established [1], [2], it is less clear whether vigorous exercise provides even greater protection from incident breast cancer [19], [20] or survival [21]–[23].

Other than age and menopause status, baseline bra cup size was the most significant risk factor for fatal breast cancer in the women runners studied here. In young, lean women, larger breast size has been occasionally associated with breast cancer risk [4], [5]. Egan et al reported that in women who were very lean when young, a larger pre-pregnancy breast size was predictive of postmenopausal breast cancer [4]. Kusano et al. reported that a larger bra cup size at age 20 was predictive of premenopausal breast cancer in the Nurses Health Study II [5]. In our cohort, the association was not limited to young-adulthood breast volume, nor was it significantly affected by age, nulliparous status, or menopausal status. The average baseline BMI of the runners and walkers who died of breast cancer was 23.78 kg/m^2^, which is leaner than the general population. In the current study, the increased risk for breast cancer mortality with breast size remained significant when adjusted for baseline circumferences of the waist, hip and chest, was not necessarily exclusive to premenopausal or postmenopausal breast cancer, and became even more significant when adjusted for baseline BMI.

Breasts consist of radiographically dense (breast epithelium and stroma) and nondense tissue (adipose tissue) whose proportions differ across women. Those whose breast are ≥75% dense tissue have a four- to six-fold higher breast cancer risk than women with <25% dense tissue [24]. On average, larger breasts contain more epithelial cells at risk. However, breast adipose tissue may be protective [25], [26], and the relative proportion of epithelial cells to protective tissue may determine risk [25]. BMI tends to be negatively correlated with the amount of dense tissue and positively correlated with the amount of protective nondense tissue [25]. In the general population, radiographic density is less of a risk factor for larger than smaller breasts [27]. In physically active women, however, breast size may be an important risk factor because the runners and walkers were generally lean, which means their large breasts tend to contain more epithelial cells at risk and less fat that may be protective.

Hormones and genetics may also contribute to the greater risk from larger breasts. Higher circulating estrogen levels are associated with greater breast cancer risk [28], and their reduction is one of the mechanisms by which physical activity is thought to lower risk [29]. Larger breasted women have significantly higher estrogen concentrations than smaller breasted women if they have narrow waists (e.g., as characteristic of physically active women) but not if they have broad waists (characteristic of sedentary women) [30]. Abdominal fat per se may suppress breast size, since its liposuction often leads to spontaneous breast enlargement [31]. High insulin-like growth factor-1 concentrations are linked to both larger breast volume [32] and increased risk of premenopausal breast cancer [33]. In addition, larger breasts tend to be more asymmetric [34], which is also a purported risk factor [35]. Breast size and breast cancer risk might also be genetically associated. Breast size heritability is about 56% [36]. Two of the seven single nucleotide polymorphisms (SNPs) associated with larger breast size have been shown to be in linkage disequilibrium with SNPs associated with greater breast cancer risk [37]. Coffee intake in women with CYP1A2*1F C-allele is associated with both smaller breast size and lower breast cancer risk in BRCA1 carriers [38].

Breast size may also affect survival. Women with breast volumes ≥850 ml have shorter disease-free and metastasis-free survival for ER-positive tumors [39]. Larger breast size also increases the odds of late-stage disease [40]. Poorer survival may explain the greater risk increase we observed for C-cup (398% vis-à-vis A-cup) and D-cup (467% vis-à-vis A-cup) mortality than the Nurses' Health Study observed for ≥D-cup (80% vis-à-vis A-cup) morbidity [5].

There are important limitations to these analyses. The date and disease stage at diagnosis and the type of breast cancer treatment are not known because diagnosis would have occurred after the baseline survey. Physical activity, bra cup size, and other baseline variables were from self-report from the participants' baseline questionnaires. Bra cup size may not be a very precise measurement of breast volume. It has been suggested that the majority (70% to 100%) of women do not wear the correct size bra [41], [42]. In particular, women tend to wear bras with bands one size too large and cups one size too small. This, however, primarily relates to issues of ideal support rather than biases that would affect associations with disease risk, it being unlikely that women would choose better-fitting bras if they were at greater breast cancer risk. Other studies have reported significant associations between breast cancer and self-reported bra cup size as an estimate of breast volume [4], [5], [43].

Exercise levels, bra size, and other subject characteristics could have changed prior to the onset of breast cancer. However, imprecision in breast volume and exercise energy expenditure based upon self-reported bra size and usual distance run are expected to attenuate their associations [12], [13]. Our results suggest that breast size in physically active women is more significantly predictive of breast cancer mortality than any other baseline risk factor except age and menopause, which may be a special consequence of the runners' and walkers' leanness, or their physically active state.
